# *Iva xanthiifolia* leaf extract reduced the diversity of indigenous plant rhizosphere bacteria

**DOI:** 10.1186/s12870-023-04316-6

**Published:** 2023-06-02

**Authors:** Jia-wen Wu, Feng-lan Li, Shu-kuan Yao, Zi-yi Zhao, Xu Feng, Rong-ze Chen, Yong-qing Xu

**Affiliations:** 1 College of Horticulture and Landscape Architecture, Northeast Agricultural University, Harbin, 150030 China; 2 College of Life Science, Northeast Agricultural University, Harbin, 150030 China; 3 Agriculture and Rural Affairs Bureau, Jinxiang, Jining, Shandong 272200 China; 4 Guangxi Institute of Chinese Medicine & Pharmaceutical Science, Nanning, 530022 China

**Keywords:** Invasive plants, Allelopathy, Rhizobacteria, Diversity, Taxonomic analysis, Bacterial function, Indigenous plants

## Abstract

**Background:**

*Iva xanthiifolia*, native to North America, is now widely distributed in northeastern China and has become a vicious invasive plant. This article aims to probe the role of leaf extract in the invasion of *I. xanthiifolia*.

**Methods:**

We collected the rhizosphere soil of *Amaranthus tricolor* and *Setaria viridis* in the invasive zone, the noninvasive zone and the noninvasive zone treated with extract from *I. xanthiifolia* leaf, and obtained *I. xanthiifolia* rhizosphere soil in the invasive zone. All wild plants were identified by Xu Yongqing. *I. xanthiifolia* (collection number: RQSB04100), *A. tricolor* (collection number: 831,030) and *S. viridis* (collection number: CF-0002-034) are all included in Chinese Virtual Herbarium (https://www.cvh.ac.cn/index.php). The soil bacterial diversity was analyzed based on the Illumina HiSeq sequencing platform. Subsequently, taxonomic analysis and Faprotax functional prediction were performed.

**Results:**

The results showed that the leaf extract significantly reduced the diversity of indigenous plant rhizosphere bacteria. *A. tricolor* and *S. viridis* rhizobacterial phylum and genus abundances were significantly reduced under the influence of *I. xanthiifolia* or its leaf extract. The results of functional prediction showed that bacterial abundance changes induced by leaf extracts could potentially hinder nutrient cycling in native plants and increased bacterial abundance in the *A. tricolor* rhizosphere related to aromatic compound degradation. In addition, the greatest number of sensitive Operational Taxonomic Units (OTUs) appeared in the rhizosphere when *S. viridis* was in response to the invasion of *I. xanthiifolia.* It can be seen that *A. tricolor* and *S. viridis* have different mechanisms in response to the invasion of *I. xanthiifolia*.

**Conclusion:**

*I. xanthiifolia* leaves material has potential role in invasion by altering indigenous plant rhizosphere bacteria.

## Background

With the development of the transportation industry and the increasingly close economic and trade exchanges among countries around the world, an increasing number of plants have been intentionally or unintentionally brought from their original habitat to new habitats for colonization. Due to the lack of natural enemies and the strong competitive ability of exotic plants, these introduced plants can spread widely and replace native plants to become invasive plants, seriously affecting biodiversity and ecosystem function stability [1–3]. Exotic invasive plants cause serious economic losses worldwide, up to $8 billion a year in the United States alone [4]. Researchers have made many guesses and hypotheses about how exotic plants might have succeeded in invading. One of the most widely accepted hypotheses is the “new weapons hypothesis” or allelopathy [5–7].

Allelopathy, as a widely studied invasion mechanism, was proposed by Molish in 1937 [8]. It was supplemented by Rise in his book Allelopathy in 1974 [9] and in the reprint of Allelopathy in 1984 [10]. Allelopathy refers to the interactions between plants [11, 12], plants and microorganisms [13, 14], and microorganisms and microorganisms [15, 16]. The modes of action include promotion and inhibition [17, 18]. Allelopathy depends on the allelopathic substances released by the root, stem, leaf and other organs of plants. The allelopathic substances in different plant organs have different intensities. Appiah KS studied the allelopathy of crude extracts from leaves, roots, inflorescences and stems of *Rosmarinus officinalis* on *Lactuca sativa*. The results showed that the crude extract of *R. officinalis* leaves had the strongest growth inhibition on various organs of *L. sativa* [19]. The release of allelochemicals can be divided into rain and mist leaching, root secretion, litter decomposition and aboveground volatilization [20]. Allelochemicals released by one pathway are said to be the main pathway when they exert the most reinforcing effect. Different plants release allelochemicals in different ways. The secondary metabolites in *Tithonia diversifolia* that have more enhanced effects are released into the soil through the decomposition of plant residues and root exudates [11]. *Eucalyptus urophylla* releases allelochemicals through rain and mist, making it difficult for native plants to grow [21].

The survival of plants depends on the sufficient space, water and nutrients provided by the soil. The microorganisms contained in the soil can transform organic nitrogen and phosphorus, which cannot be directly absorbed and utilized by plants, into an inorganic state that can be absorbed [22]. There has recently been increasing evidence that many organisms (particularly fungi and bacteria) play important roles in soil nutrient cycling. For example, when soil organic matter content increases, it leads to an increase in microbial biomass, which stimulates carbon and nitrogen cycling in the soil [23]. Phosphate solubilizing and potassium solubilizing bacteria metabolize phosphorus and potassium in soil that plants cannot use directly. While satisfying their own life needs, they also decompose these substances into ionic states that can be directly absorbed by plants [24–26]. Soil microorganisms also play an important role in the degradation of organic pollutants in soil. Studies have shown that Bacteroidetes and Proteobacteria have a strong ability to degrade and remove soil organic pollutants [27, 28]. Firmicutes have a certain decomposition ability for various soil organic pollutants [29]. Thus, soil microorganisms have a great impact on plant-dependent soil and indirectly affect the living conditions of plants by influencing the soil environment.

While soil microorganisms play a key role in the growth, development and reproduction of plants, plants also provide root exudates, litter and other nutrients for soil microorganisms to metabolize and utilize to maintain life. Different plant root exudates may lead to the specificity of soil microbial structures enriched in plant roots [30]. Plant-soil microorganisms influence, promote and restrict each other, and they promote the succession of communities in dynamic changes. The balance of plant-soil-soil microbe dependency is broken when one of its members changes due to certain influencing factors. Alien plant invasion is a common and important factor that causes significant changes in indigenous plants and indigenous rhizosphere soil microorganisms [31, 32]. Niu et al. studied the structure of the soil microbial community in the invasion area of *Ageratina adenophora*. The results showed that the fungal content in the invasion area increased obviously after invasion [33]. Vitousek et al. studied the reasons for the successful invasion of *Rhus typhina* into a low-nitrogen environment. *R. typhina* can enrich nitrogen-fixing bacteria and increase the nitrogen content in soil to compete with indigenous plants for space and resources in a low-nitrogen environment to ultimately achieve successful invasion [34]. Thus, alien plants can change the structure of the soil microbial community in the invasion area to make it suitable for their own survival but often have adverse effects on the survival of local indigenous plants and promote the succession of the biological community in a certain direction.

*Iva xanthiifolia* (Asteraceae) was transferred from North America to China by commercial trade along with cargo transportation. After its introduction, it colonized, spread and excluded native plants in China, becoming an invasive alien plant that occupies a wide ecological niche. At present, studies on the successful invasion of *I. xanthiifolia* have focused on the effect of allelopathy of *I. xanthiifolia* on recipient plants. Studies have shown that extracts from various parts of *I. xanthiifolia* have allelopathic effects on seed germination and seedling growth of recipient plants [35]. Leaves are the organs that exert the most allelopathy [36]. Leaf extracts at low concentrations inhibited seed germination and seedling growth of *Brassica juncea*, *Brassica oleracea*, *A. tricolor*, and *S. viridis* [36, 37]. The main components in the leaf extract were analyzed by gas chromatography-mass spectrometry, and the potential allelochemicals were found to be terpenoids and their derivatives (2-Camphor, Borneol, 1-Caryophyllene, and Ragnidin) and phenolic acids (Isovanillin, 2-Methallylphenol) [37]. However, there are few studies on the indirect effects of *I. xanthiifolia* on soil microorganisms. Therefore, this paper used high-throughput sequencing technology to investigate the effects of *I. xanthiifolia* and leach extracts from *I. xanthiifolia* leaves on the rhizosphere soil bacteria of recipient plants, aiming to provide a new theoretical basis for the study of the allelopathy mechanism of *I. xanthiifolia*.

## Results

### Alpha diversity analysis

**Fig. 1:**
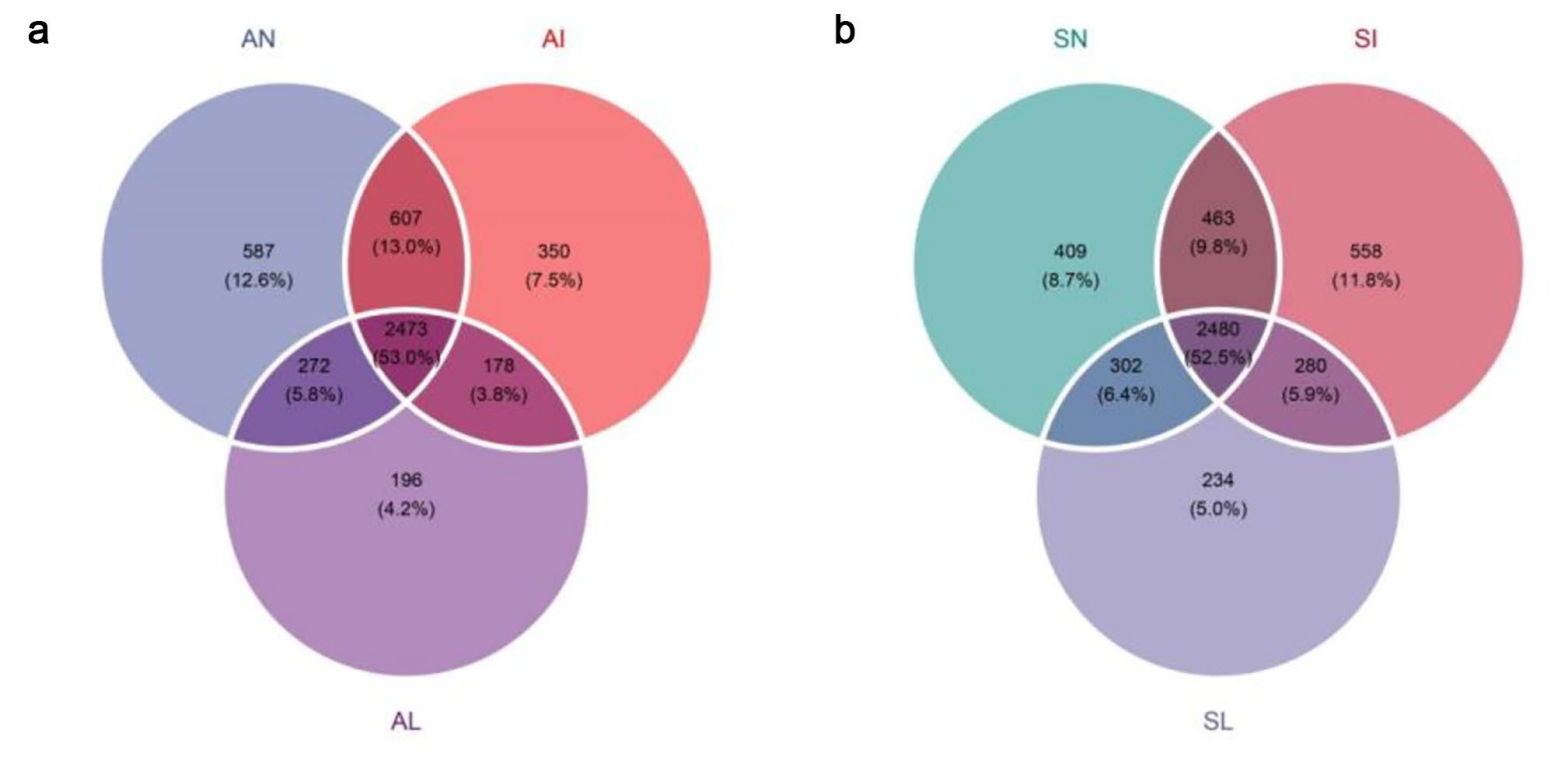
OTU analysis. **(a)** Common and unique OTUs of *A. tricolor* rhizobacteria in noninvasive area (AN), invasive area (AI), and leaf extract treated area (AL). **(b)** Common and unique OTUs of *S. viridis* rhizobacteria in the noninvasive area (SN), invasive area (SI), and leaf extract treated area (SL)

**Fig. 2:**
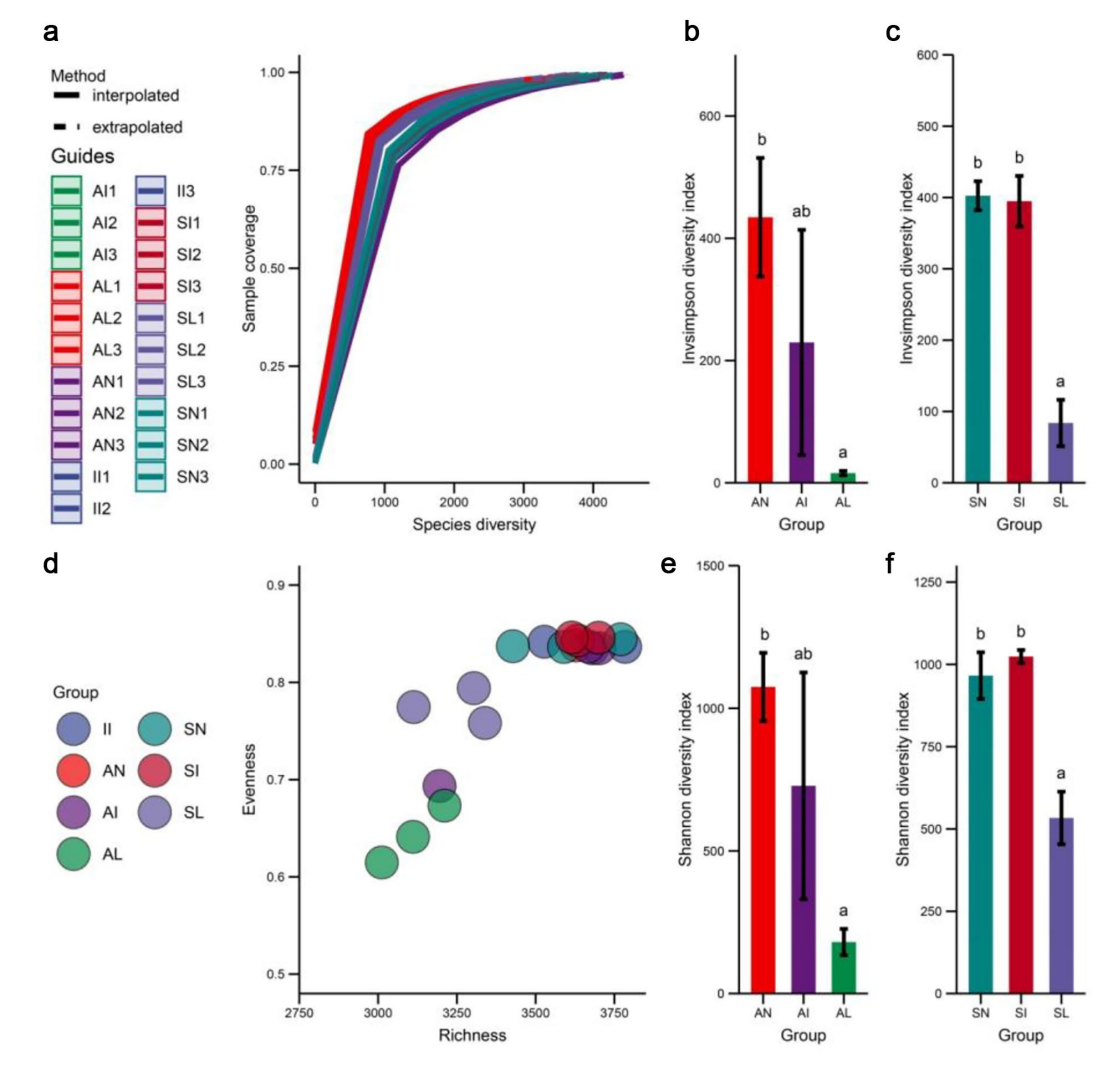
Alpha diversity analysis. II stands for *I. xanthiifolia* rhizobacteria in invasive area. AN, AI, and AL represent *A. tricolor* rhizobacteria in noninvasive area, invasive area, and leaf extract treated area, respectively. SN, SI, and SL represent *S. viridis* rhizobacteria in noninvasive area, invasive area, and leaf extract treated area, respectively. **a** Dilution curves. **b-c**. Inverse Simpson index. **d**. Species richness and evenness analysis. e-f. Shannon diversity index. Different letters represent significant differences (Tukey HSD test, *P* < 0.05)

The high-throughput sequencing data showed that there were 1,368,907 clean tags in 21 samples. At the level of similarity above 97%, there are 5547 OTUs present in the dataset. In different treatment areas, there were 2473 (53%) shared OTUs in the rhizosphere of *A. tricolor* and 2480 (52.5%) shared OTUs in the rhizosphere of *S. viridis*. The unique OTUs analysis results showed that the unique OTUs of *A. tricolor* and *S. viridis* in the leaf extract treatment area were 196 (4.2%) and 234 (5%), respectively, which were the lowest in each treatment area (Fig. 1). In this study, a certain number of individuals were randomly selected from the soil bacteria data set of 21 samples of 7 types, and the number of species represented by these individuals was counted, and the dilution curve was constructed. The results showed that 98.1 − 98.3% coverage represented 3011.981–3930.983 OTUs, which were basically consistent with the observed values (3012–3931) (Fig. 2a). This represents that the measure depth meets the needs of in-depth analysis. The rhizosphere bacterial species richness of *A. tricolor* and *S. viridis* treated with the aqueous extract of *I. xanthiifolia* leaves was lower than that in the natural growth state (Fig. 2a, d). In order to better characterize whether the bacterial community diversity was significantly changed after the treatment, the Shannon diversity index (Fig. 2e, f) and the Inverse Simpson index (Fig. 2b, c) were statistically analyzed. The analysis results showed that the Shannon diversity index and Inverse Simpson index of *A. tricolor* and *S. viridis* were significantly down-regulated after treatment with leaf extract compared with indigenous plants that formed a population scale (Tukey HSD test, *P* < 0.05). However, there was no significant change in the bacterial community diversity index of indigenous plants in the invasive zone (Tukey HSD test, *P* > 0.05). This demonstrates that the rhizosphere bacterial diversity of *A. tricolor* and *S. viridis* is sensitive to the aqueous extract of *I. xanthiifolia* leaves. This explanation is supported by the reduced rhizosphere bacterial evenness of *A. tricolor* and *S. viridis* treated with the leaf extract (Fig. 2d), which also indicates that the number of individuals within the species in the community was also changing.

### Beta diversity analysis

**Fig. 3:**
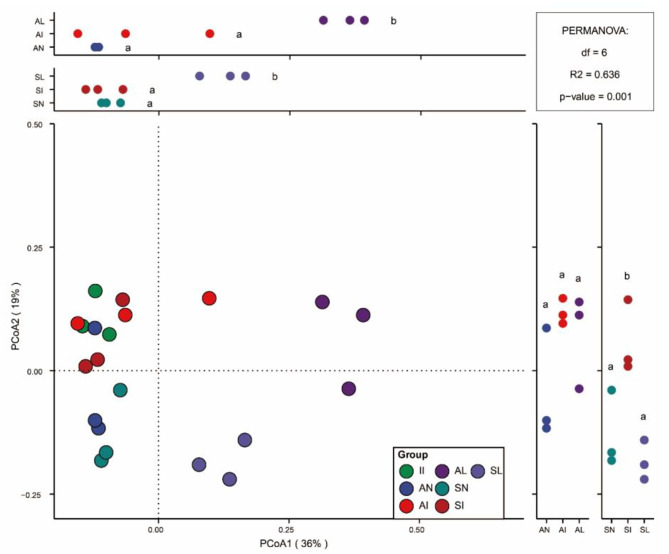
Bray-Curtis-based Beta diversity analysis. II stands for *I. xanthiifolia* rhizobacteria in invasive area. AN, AI, and AL represent *A. tricolor* rhizobacteria in noninvasive area, invasive area, and leaf extract treated area, respectively. SN, SI, and SL represent S. viridis rhizobacteria in noninvasive area, invasive area, and leaf extract treated area, respectively. Different letters represent significant differences (Tukey HSD test, *P* < 0.05)

The bacterial community differences were quantified by calculating the distance matrix by dimensionality reduction to analyze the degree of differentiation between biological communities and the relationship between diversity distances between different samples. We performed principal coordinate analysis of rhizobacterial OTUs of *I. xanthiifolia* and native plants in different treatment areas based on the Bray-Curtis weighted distance method (Fig. 3). The first principal coordinate provides 36% variance contribution, and the second principal coordinate provides 19% variance contribution. Differences between different treatment groups were significant (PERMANOVA: R^2^ = 0.636, *P* = 0.001). In order to more clearly present the degree of differentiation of bacterial communities in the samples, the first and second principal coordinates were analyzed for significance of difference based on the Turkey HSD test method. In the first principal coordinate difference comparison, the indigenous plants in the leaf extract treatment area were significantly different from those in the noninvasive and invasive areas (Turkey HSD Test, P < 0.05). This indicated that the leaf extract had strong effect on the rhizosphere bacterial community structure of indigenous plants.

### Sensitive species analysis

**Fig. 4:**
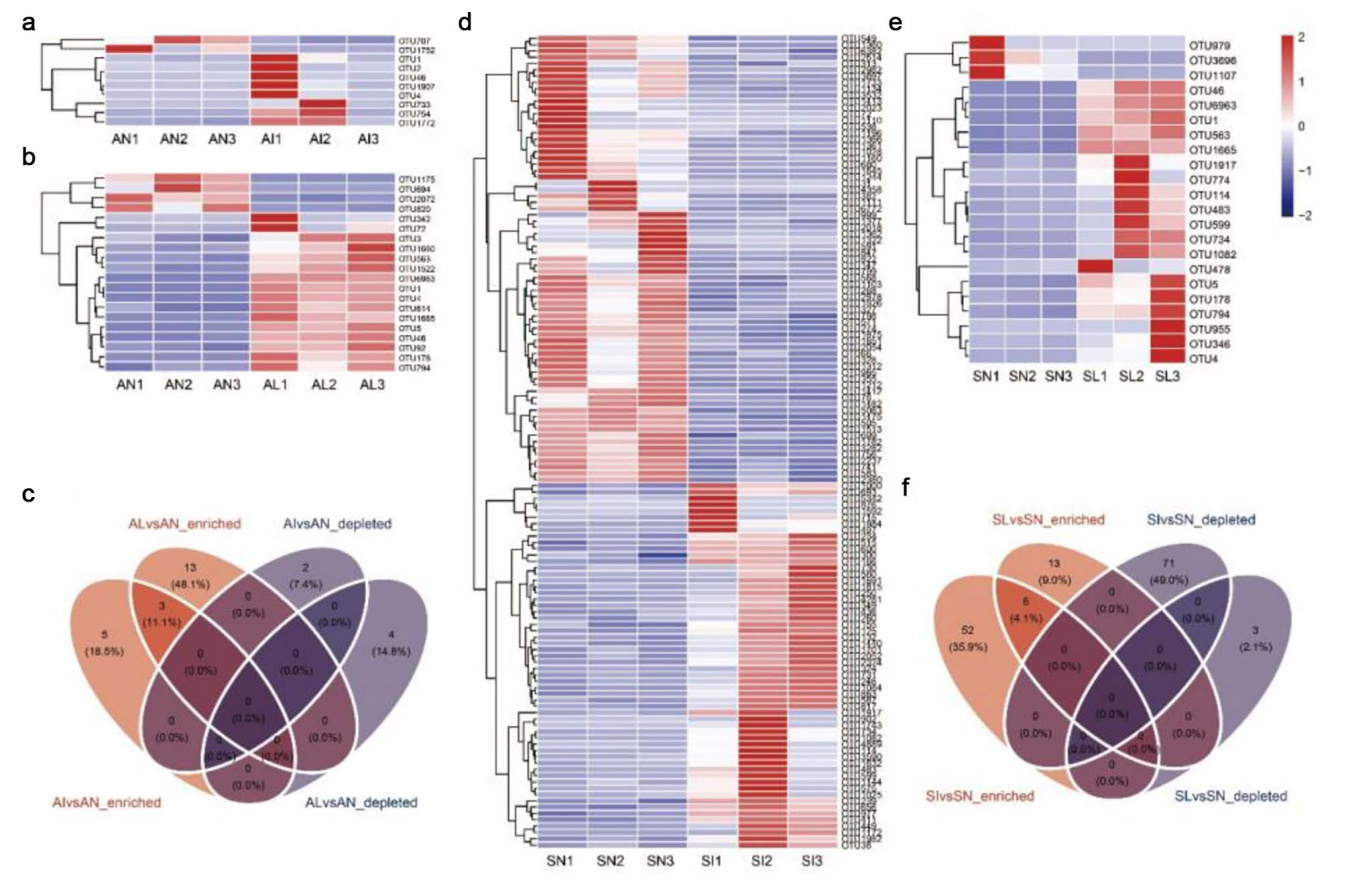
Differential OTU analysis. AN, AI, and AL represent *A. tricolor* rhizobacteria in noninvasive area, invasive area, and leaf extract treated area, respectively. SN, SI, and SL represent *S. viridis* rhizobacteria in noninvasive area, invasive area, and leaf extract treated area, respectively. Compared with the noninvasive area, the difference OTUs of the *A. tricolor* rhizosphere in the invasive area **(a)** and the leaf extract treatment area **(b)** were analyzed. Compared with the noninvasive area, the difference OTUs of *S. viridis* rhizosphere in the invasive area **(d)** and the leaf extract treatment area **(e)** were analyzed. Red represents a significant increase (Kruskall Wallis test, logFC > 1 & *P* < 0.05) and blue represents a significant decrease (Kruskall Wallis test, logFC < 0.5 & *P* < 0.05). Significantly enriched and reduced OTUs were counted **(c, f)**

Indigenous plant rhizosphere bacteria OTUs had different degrees of response to the invasion of *I. xanthiifolia* or its leaf extract. Indigenous plant rhizobacterial OTUs varied in the extent to which they respond to *I. xanthiifolia* invasion or their leaf extracts. There were a total of 129 sensitive OTUs in the rhizosphere of *S. viridis* in the invasion area (Fig. 4d, f), which was the largest number of different OTUs in each treatment compared with the noninvasion area. There were 22 sensitive OTUs in *S. viridis* in the leaf extract treatment area (Fig. 4e, f). In addition, the sensitive OTUs of *A. tricolor* in the invasion area and the leaf extract treatment area were 10 and 20, respectively (Fig. 4a, b, c). There were 3 sensitive OTUs that were consistently up-regulated in *A. tricolor* in the invasion zone and the leaf extract treatment zone (Fig. 4c). There were 6 sensitive OTUs that were consistently up-regulated in *S. viridis* in the invasion zone and the leaf extract treatment zone (Fig. 4f).

**Fig. 5:**
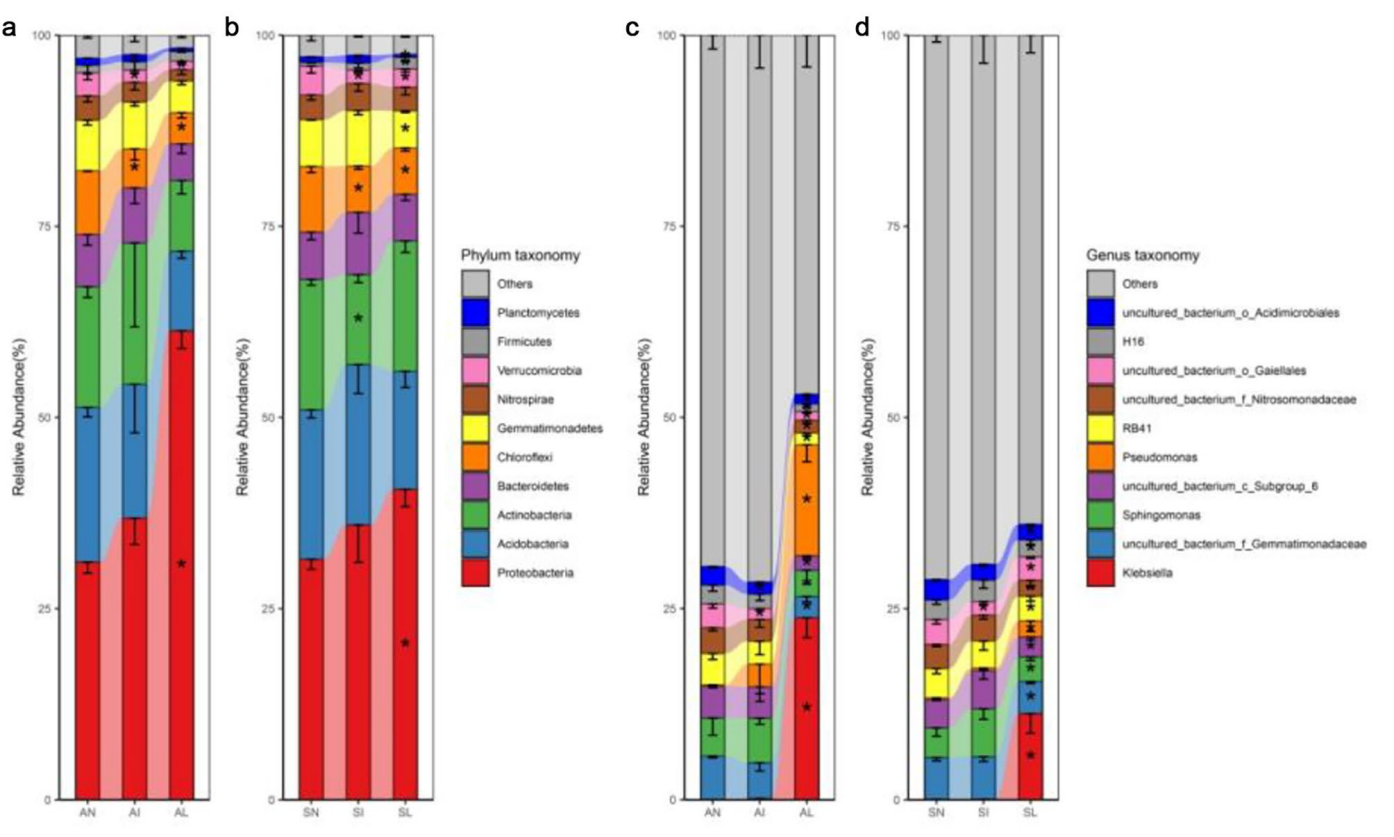
Analysis of differential phyla **(a, b)** and genera **(c, d)**. AN, AI, and AL represent *A. tricolor* rhizobacteria in noninvasive area, invasive area, and leaf extract treated area, respectively. SN, SI, and SL represent *S. viridis* rhizobacteria in noninvasive area, invasive area, and leaf extract treated area, respectively. The error bars only show the negative semi-axis for easy viewing. “*” represents a significant difference, marked on the column of the experimental group (Kruskall Wallis test, *P* < 0.05)

The relative abundance of species was analyzed at the phylum level, and it was found that Proteobacteria, Acidobacteria, Actinobacteria, Bacteroidetes, Chloroflexi, and Gemmatimonadetes were the main phyla (relative abundance ratio > 5%). The relative abundance analysis of species at the genus level showed that *Klebsiella*, uncultured_bacterium_f_Gemmatimonadaceae, *Sphingomonas*, uncultured_bacterium_c_Subgroup_6, *Pseudomonas* and RB41 were the main genera (relative abundance ratio > 3%). Based on the method of Kruskal-Wallis test, the difference significance analysis of the sequence entries of different phyla and genera of indigenous plant rhizosphere bacteria in different treatment areas was carried out. The results showed that the abundance of Chloroflexi and Verrucomicrobia in the rhizosphere of indigenous plants in both the invasive and leaf extract-treated areas decreased significantly compared with the noninvasive areas (Kruskal-Wallis test, *P* < 0.05) (Fig. 5a, b ). *I. xanthiifolia* leaf extract significantly increased the abundance of Proteobacteria in the rhizosphere of indigenous plants (Kruskal-Wallis test, *P* < 0.05) (Fig. 5a, b). The genus-level analysis showed that the abundance of uncultured_bacterium_o_Gaiellales and uncultured_bacterium_o_Acidimicrobiales in both the invasion area and the leaf extract treatment area were significantly decreased (Kruskal-Wallis test, *P* < 0.05) (Fig. 5c, d). In addition, the leaf extract significantly altered the top ten bacteria in relative abundance, and significantly increasing *Klebsiella* spp. and *Pseudomonas* spp. in the rhizosphere of *A. tricolor* and *S. viridis* (Kruskal-Wallis test, *P* < 0.05) (Fig. 5c, d). It indicates that the invasion of *I. xanthiifolia* or short-term foreign substances has a strong interference on the bacterial community structure, and can recruit or repel bacteria of specific genera, and the response of recipient plants to it is universal.

### Functional prediction

**Fig. 6:**
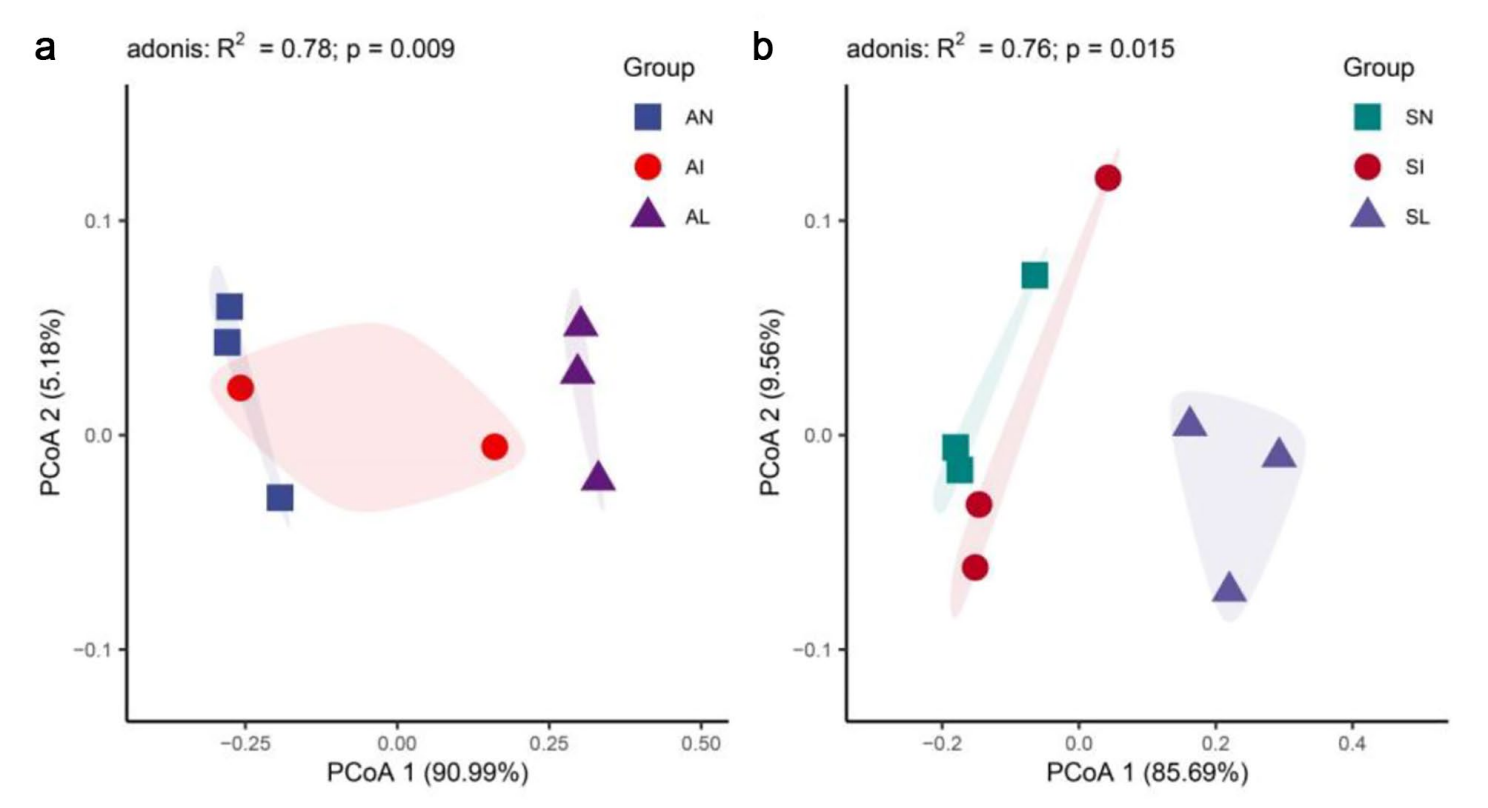
Principal coordinate analysis of predicted functions. AN, AI, and AL represent *A. tricolor* rhizobacteria in noninvasive area, invasive area, and leaf extract treated area, respectively. SN, SI, and SL represent *S. viridis* rhizobacteria in noninvasive area, invasive area, and leaf extract treated area, respectively

This experiment predicted the function of rhizosphere bacterial samples. 1167 OTUs were classified into 52 functions. Chemoheterotrophy, aerobic_chemoheterotrophy, fermentation, nitrate_reduction and nitrification were the main functions of soil bacterial samples (relative abundance > 5%). The results of principal coordinate analysis showed that *A. tricolor* was significantly different in different treatment areas (adonis: R^2^ = 0.78, *P* = 0.009), as was *S. viridis* (adonis: R^2^ = 0.76, *P* = 0.015). The PCoA1 contributions of *A. tricolor* and *S. viridis* were 90.99% and 85.69%, respectively (Fig. 6a, b).

**Fig. 7:**
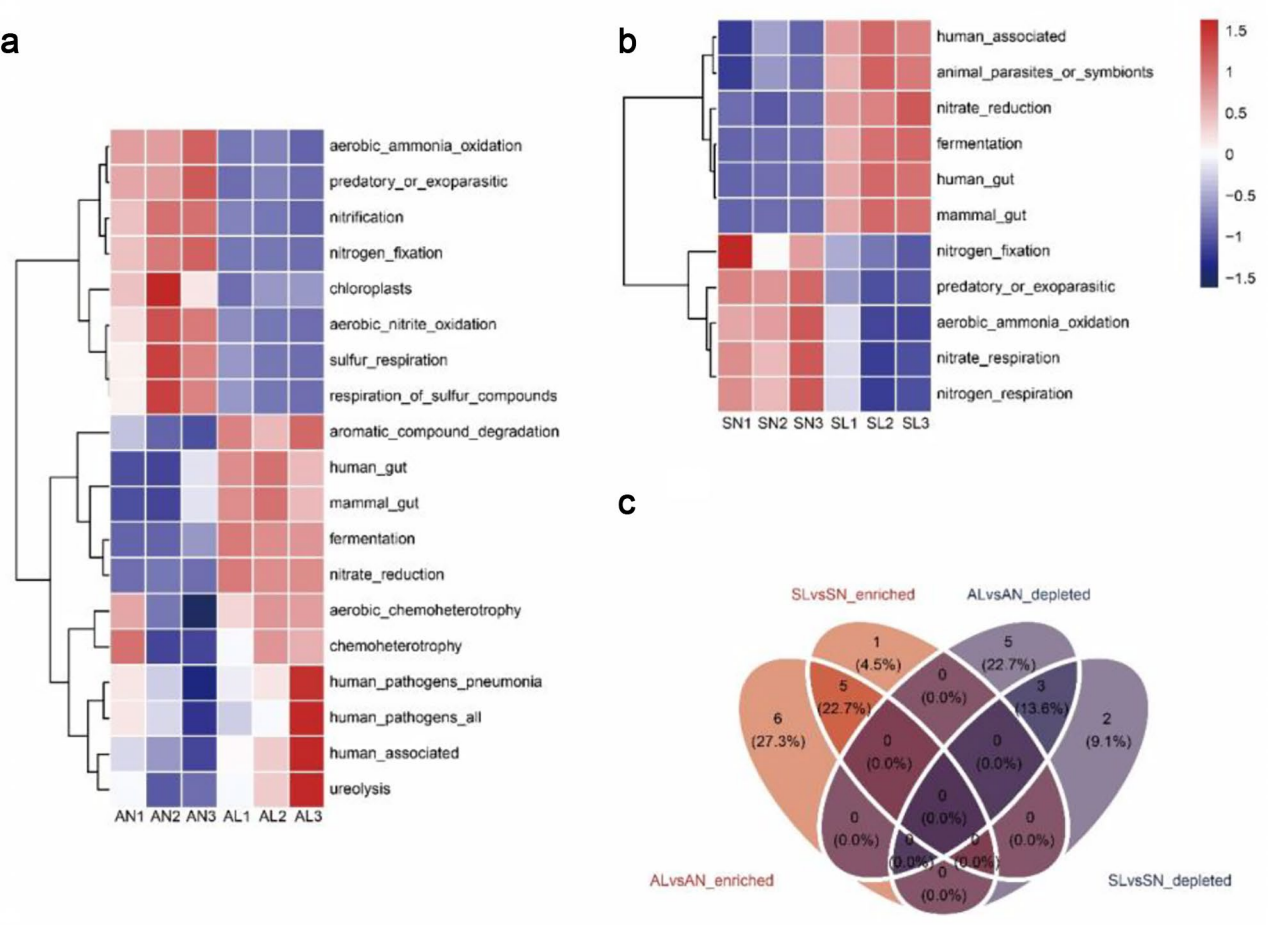
Differential functional analysis. **(a)** Differential function of *A. tricolor* in *I. xanthiifolia* leaf extract treatment area and noninvasive area. **(b)** Differential function of *S. viridis* in *I. xanthiifolia* leaf extract treatment area and noninvasive area. Red represents a significant increase (Kruskall Wallis test, logFC > 1 & *P* < 0.05) and blue represents a significant decrease (Kruskall Wallis test, logFC < 0.5 & *P* < 0.05). **(c)** Functional analysis of significant enrichment and reduction of both common and specific

The leaf extract significantly reduced the nitrogen cycling function of native plants, and the results are shown in Fig. 7. The abundance of nitrogen fixation and aerobic ammonia oxidation in *A. tricolor* and *S. viridis* rhizobacteria were significantly reduced (Kruskal-Wallis test, *P* < 0.05, Fig. 7a and b). In addition, the abundance of nitrification and aerobic nitrite oxidation in the rhizosphere of *A. tricolor* and the abundance of nitrate respiration and nitrogen respiration in the rhizosphere of *S. viridis* were significantly decreased (Kruskal Wallis test, *P* < 0.05, Fig. 7a and b). The sulfur cycling potential of *A. tricolor* rhizosphere bacteria was also hindered by the leaf extract. The abundances of sulfur respiration and respiration of sulfur compounds were significantly reduced (Kruskal-Wallis test, *P* < 0.05, Fig. 7a). However, the abundance of aromatic compound degradation, aerobic chemoheterotrophy and chemoheterotrophy in the rhizosphere of *A. tricolor* was significantly increased (Kruskal Wallis test, *P* < 0.05, Fig. 7a). The abundance of bacteria related to energy production by fermentation was also significantly increased in the rhizosphere of indigenous plants (Kruskal Wallis test, *P* < 0.05, Fig. 7a and b). The same 5 functions (human_gut、fermentation、mammal_gut、nitrate_reduction、human associated, Fig. 7a and c) were significantly up-regulated, and the same 3 functions (predatory_or_exoparasitic、aerobic ammonia oxidation、nitrogen_fixation, Fig. 7b and c) were significantly down-regulated in the indigenous plants of the leaf extract treatment area and the non-invasion area.

## Discussion

When an alien plant enters a new habitat and experiences a long or short interaction with an indigenous plant, the end result may be symbiosis, becoming a single dominant species, or failure to colonize in the new habitat and being replaced by an indigenous plant [38, 39]. Foreign plants that successfully colonize, especially those that become a single dominant species, are called invasive plants. The reasons for the successful invasion of exotic plants are various, and most of the known factors point to the allelopathy caused by the secondary metabolites released or secreted by exotic plants on indigenous plants and their root microbial communities [1]. The secondary metabolites have aggregation effects on soil bacteria, and the secondary metabolites released by local plants accumulate for a long amounts of time, attracting a large number of soil bacteria [40, 41]. These soil bacteria form a relatively stable symbiotic relationship with native plants. After entering the new habitat, foreign plants can change soil microbial species, flora number, community structure and diversity, and destroy the equilibrium symbiosis formed by long-term evolution between indigenous plants and soil microorganisms [42, 43]. After a temporary period of instability, the soil bacteria become stable again under the interaction of exotic plants- and soil-native plants [44]. It is only a matter of time before indigenous plants are replaced when the resulting new balance is more in favorfavour of exotic plants [45].

In the present study, the alien plant invasion did not have significant effects on the diversity of rhizosphere bacteria in indigenous plants, but some OTUs significantly responded to alien plants resulting in changes in relative abundance in the microbiota. Changes in the rhizosphere bacterial operative taxa of *S. viridis* were particularly prominent in the invasion zone (Fig. 4d, f). This may be due to the differences in the ability of different indigenous plants to recruit rhizosphere bacteria under invasion conditions, and the weak bacterial flora stability is susceptible to perturbation by the invading plants [46]. This result is a supplement to the published content of our research group. Previous work found that both *A. tricolor* and *S. viridis* sensitively responded to the extract of *I. xanthiifolia* leaves. The seed germination index and seedling growth index decreased significantly, but the comprehensive allelopathic effect index of *A. tricolor* affected by the leaf extract was the lowest [36]. From this, it can be seen that the succession mechanism of *I. xanthiifolia* for indigenous plants has certain diversification and bias according to plant species and characteristics.

Plants and soil bacteria are interdependent and restrict each other. While plants provide nutrients to soil microbes, they also selectively recruit soil microbes to gather in the rhizosphere [49]. This study found that Prteobacteria, Acidobacteria, and Actinobacteria were the dominant phyla within the scope of the test. These bacteria are involved in the cycling of nutrients such as carbon, nitrogen and sulfur in the soil [47–49]. Adequate nutrients can increase the accumulation of organic matter in plants and promote good plant growth. In this study, the treatment of *I. xanthiifolia* leaf extract significantly increased the abundance of Proteobacteria in the rhizosphere soil of *A. tricolor* and *S. viridis*. Proteobacteria are one of the most abundant bacteria in soil [50]. However, it could not be concluded that the leaf extract treatment improved nutrient cycling in the soil. Because the newly emerging higher relative abundance of Klebsiella at the genus level was the main reason for the increase in the relative abundance of the native plant Proteobacteria in the leaf extract treated area. *Klebsiella* spp. can cause pomegranate fusarium wilt [51]. It has also been reported that *Klebsiella* spp. has a growth-promoting effect on *Dianthus* caryophyllus [52] and tomato [53]. Therefore, the impact of each Klebsiella species on different indigenous plants needs to be verified by subsequent studies. Different indigenous plants in the invasive zone were affected by invasive plants, and phyla (Verrucomicrobia, Chloroflexi) and genera (uncultured_bacterium_o_Acidimicrobiales, uncultured_bacterium_o_Gaiellales) showed a significant decrease in consistency (Fig. 5c, d). These microorganisms are considered probiotics in numerous reports. Verrucomicrobia has been reported to be able to participate in the construction of rhizosphere bacterial communities and establish a good interaction between microorganisms and plants [54]. Verrucomicrobia and Chloroflexi have been reported to significantly enrich soil quality in response to biochar treatment [55]. Because the leaf extract of *I. xanthiifolia* can reduce the diversity of rhizosphere bacteria of indigenous plants. Indigenous plants may release secretions to maintain bacterial flora stability or recruit beneficial bacteria, which will consume more carbon sources. In this experiment, *Pseudomonas* spp. and *Klebsiella* spp. significantly increased in the rhizosphere of indigenous plants after treatment with leaf extract, but no similar phenomenon appeared in the symbiotic zone. Pseudomonas can secrete phenazine, through which it is resistant to many plant pathogens such as fungi, bacteria and oomycetes, and is a widely reported plant probiotic [56]. This may be that the leaf extract stimulated the resistance of native plants to *Klebsiella* spp. and other bacteria that have inhibitory effects on plant growth and development. The rhizosphere soil bacterial diversity showed a significant downward trend when the indigenous plants were irrigated with leaf extract (Fig. 2a, d). The diversity of soil microorganisms in the rhizosphere is often positively correlated with plant growth [57, 58] and disease resistance [59]. Therefore, the material in the leaves of pseudoxanthium may slow down the development of indigenous plants by reducing the diversity of soil bacteria, so as to trigger the decline of plant disease resistance. Therefore, the material in the leaves of *I. xanthiifolia* may slow down the development of indigenous plants by reducing the diversity of soil bacteria, so as to trigger the decline of plant disease resistance.

*I. xanthiifolia* has a large biomass, and the long-term continuous input of substances into the soil after withering can cause strong disturbance to the soil. The leaf extract treatment resulted in enhanced function of *A. tricolor* and *S. viridis* rhizobacteria to degrade compounds. In the results, the aromatic compound degradation ability of *A. tricolor* rhizosphere was enhanced (Fig. 7a). Aromatic compounds are considered as pollutants in soil [60], and flavonoids [61] and terpenoids [62] in them can inhibit plant growth. This indirectly indicates that there may be aromatic compounds in the leaves of *I. xanthiifolia*. The identification of these substances and their effects on indigenous plants requires subsequent experimental evaluation. We also found that the leaf extract resulted in a decrease in the functional abundance of soil N and S cycling, with a repulsive effect on bacteria involved in nutrient cycling. Changes in soil bacterial function may have indirect hindrance and legacy effects on nutrient uptake and utilization of indigenous plants [63].

## Conclusion

Compared with *A. tricolor* in the invasion zone of *I. xanthiifolia*, the rhizosphere bacteria of *S. viridis* are more sensitive to *I. xanthiifolia*. *I. xanthiifolia* leaf extract reduced the rhizospheric bacterial diversity and nutrient cycling functional bacterial abundance of *A. tricolor* and *S. viridis*, and significantly increased the abundance of *Klebsiella* spp. and *Pseudomonas* spp. During the invasion of *I. xanthiifolia*, leaf-altered indigenous plant rhizosphere bacteria are of great significance to the spread of *I. xanthiifolia*.

## Materials and methods

### Obtaining the extract of I. xanthiifolia leaves

We collected mature leaves from the middle part of the *I. xanthiifolia* and removed wilted, insect- and pathogen-infested leaves in August 2020. After the leaves were dried in the shade, they were fully ground in a wall breaker. The product was passed through an 80-mesh sieve, and the residue of petioles or veins was removed to obtain dry powder. An appropriate amount of distilled water was added to accurately weighed 40 g of leaf powder, Ultrasonic treatment was performed at room temperature for 30 min to dissolve the leaf material rapidly and fully in water. The mixture was taken out and added to a Buchner funnel covered with three layers of filter paper, and filtered under reduced pressure using a vacuum filter. A 0.2 μm filter was used to remove microorganisms from the extract. Sterilized distilled water was added to the filtrate and the volume was adjusted to 1 L to obtain a treatment solution of 40 g leaf extract·L^− 1^. To avoid material loss, the obtained leaf extract was applied to the soil within 30 min. The contents of dissolved organic carbon (DOC) and dissolved organic nitrogen (DON) in leaves were 61.113 µg·g^− 1^ and 5.563 mg·g^− 1^, respectively.

### Sampling site overview, processing and sample collection

Soil samples were collected by a random five-point sampling method in September 2020. The sampling site was the abandoned wasteland around Northeast Agricultural University (45°44’50.17"N, 126°44’7.51"E; 45°44’41.83"N, 126°43’44.5"E; 45°43’36.15"N, 126°41’9.87"E). In each plot, there was an area where the *I. xanthiifolia* and *A. tricolor* / *S. viridis* grow together, and *A. tricolor* / *S. viridis* grows alone. All the weeds in the sampling sites were identified by Xu Yong-qing. The digitized specimens of plants involved in the research can all be retrieved on the Chinese Virtual Herbarium (https://www.cvh.ac.cn/index.php). The collection numbers of *I. xanthiifolia*, *A. tricolor* and *S. viridis* are RQSB04100, 831,030 and CF-0002-034, respectively. Since this experiment was carried out in a natural environment, the distribution of various plants was non-uniform. We defined the invasion area of *I. xanthiifolia*, that is, the collective name of the sampling points with an area of 1 m^2^ where *I. xanthiifolia* is adjacent to *A. tricolor*/*S. viridis*, and the distance from the stem base is less than 40 cm. A circular area with a radius of 40 cm was set as the core sampling area with the *I. xanthiifolia* as the center. The non-invasive area was a 1 m×1 m square area where *A. tricolor*/*S. viridis* thrived, which was guaranteed to be free of *I. xanthiifolia*. The invasive area and the non-invasive area were separated by 3 m or more to ensure that the non-invasive area was completely unaffected by the *I. xanthiifolia*. The non-invasion area where 1 L 40 g leaf extract/L was evenly irrigated was the leaf extract treatment area. Equal amount of sterilized distilled water was added to other areas. The core sampling area of the non-invasion area and the leaf extract treatment area was a circular area with a radius of 40 cm with the center of the sampling point as the center of the circle. There were at least 8 sampling points in each plot with different treatments (i.e., *A. tricolor*/*S. viridis* in the invasive area, non-invasion area, and leaf extract treatment area, and *I. xanthiifolia* in the invasive area), and we marked the sampling points. At 72 h after leaf extract treatment, 5 sampling points were randomly selected for each treatment in each plot, and the core sampling area of each sampling point was sampled. We removed the surface litter and other impurities, used a shovel to dig out the plants with bulk soil and rhizosphere soil, and the sampling depth was 0–25 cm soil layer from the surface. We randomly selected 3 plants corresponding to the markers and packed them into sterile collection bags. We used a biological sample sampling box with ice packs for cryopreservation of the samples during transfer. The tools used for sampling (shovels, sampling bags, etc.) were sterilized before use. We shake off the bulk soil attached to the plant roots in the ultra-clean workbench, and use a soft brush to gently brush off the rhizosphere soil with a thickness of about 1 mm attached to the roots. The rhizosphere soil of 3 plants in each core sampling area was mixed together. Plant rhizosphere soil from selected core sampling areas of the same treatment (i.e., invasive, non-invasive, and leaf extract treated areas of *A. tricolor*/*S. viridis*, and *I. xanthiifolia* from invasive areas) were mixed separately per plot. Therefore, there were three biological replicates per treatment in this experiment.

### PCR amplification and sequencing

DNA was extracted from 0.25 g of soil using a soil genomic DNA extraction kit (DP336, Tiangen Company, Beijing, China) according to the instructions. The universal primers 338F: 5’-ACTCCTACGGGAGGCAGCA-3’ and 806R: 5’-GGACTACHVGGGTWTCTAAT-3’ were used to amplify the V3-V4 region of the 16 S rRNA gene. The amplification reaction kit was Kapa Taq Extra PCR kit (Merck Group, USA). The amplification reaction kit was Kapa Taq Extra PCR kit (Merck KGaA, Darmstadt, Germany). After an initial denaturation at 95℃ for 5 min, an amplification was performed by 30 cycles of incubations for 30 s at 95℃, 20 s at 58℃, and 6 s at 72℃, followed by a final extension at 72℃ for 7 min. Then the amplified products were purified and recovered using 1.0% agarose gel electrophoresis method. Finally, the library construction and sequencing steps were performed by Beijing Biomarker Technologies Co.Ltd. All the sequencing raw data have been submitted to the BioProject at the National Center for Biotechnology Information (NCBI) with accession no. PRJNA877400.

### Bioinformatics analysis

The bioinformatics analysis in this study was completed on the Biomarker biocloud platform (www.biocloud.org). To obtain the raw tags, paired-end reads were merged using FLASH (v1.2.7, http://ccb.jhu.edu/software/FLASH/) [64]. Then, raw tags were filtered and clustered in the next steps. The merged tags were compared to the primers, and the tags with more than six mismatches were discarded by the FASTX-Toolkit [65]. Tags with an average quality score < 20 in a 50-bp sliding window were truncated using Trimmomatic (http://www.usadellab.org/cms/?page=trimmomatic) [66], and tags shorter than 350 bp were removed. We identified possible chimeras by employing UCHIME [67], a tool included in mothur (http://drive5.com/uchime) [68]. The denoised sequences were clustered using USEARCH (version 10.0) [69], and tags with similarity > = 97% were regarded as OTUs. Taxonomy was assigned to all OTUs by searching against the Silva databases (Release128, http://www.arb-silva.de.) [70] using UCLUST [71] within QIIME [72].

### Statistical analysis

Statistical analysis of all data in this study was done using the software “R” (v3.6.2, http://www.r-project.org/). We used the filter function and summarise function in the R package dplyr to filter and count two or more OTUs whose abundance is not “0” in the processing, and used the R package ggvenn to draw Venn diagrams based on the common OTUs and unique OTUs of each treatment. The alpha diversity analysis of 16s bacterial rDNA was based on the OTU abundance table after flattening by the rrarefy function in the R package vegan. The R package iNEXT was used to calculate the sample coverage based on the interpolated and extrapolated methods, and also obtained the cumulative number of species, the Shannon diversity index and the Inverse Simpson index. The R package ggiNEXT was used to draw the sample dilution curve according to the sample coverage [73]. The diversity function of the R package vegan was used to calculate Shannon.Wiener, and the evenness was calculated according to the formula Shannon.Wiener/log(cumulative number of species (species richness)) [74].

The vegdist function in the R package vegan was used to process the OTU table and build the dissimilarity distance matrix based on the Bray-Curtis method (999 permutations). The principal coordinate analysis (pcoa) results were obtained by using the pcoa function in the R package ape, and PCoA1 and PCoA2 were extracted to draw a scatter plot through ggplot2. We performed one-way ANOVA for PCoA1 and PCoA2 using the aov function in the R package multcomp, respectively, and then used the glht function to perform pairwise comparisons based on Tukey HSD test. The cld function was used to extract letter labels representing differences between groups, and ggplot2 was used to draw individual observations chart. We performed Permutational multivariate analysis of variance (PERMANOVA) by using the adonis function in the R package vegan based on the Bray-Curtis method (999 permutations) [75]. The obtained results were presented together with individual observations chart and scatter plots. We filtered low-abundance OTUs with relative abundances < 0.005%, and comparisons of OTUs between groups were performed by the R package edgeR [76, 77]. We used the DGEList function to transform the OTU abundance table, and then used the calcNormFactors function to normalize the data. Fold change and p-value were obtained after comparison between groups using glmLRT. logFC > 1&*P* < 0.05 means significant enrichment, logFC < 0.5 & *P* < 0.05 means significant reduction. We also used the decideTestsDGE function for p-value correction based on the FalseDiscovery Rate method ,and controlled for false discovery rate probability of less than 5%. The R package pheatmap was used for the difference OTUs clustering heatmaps between the two groups.

Analysis of major bacterial phyla and genera composition was performed using the R package. The abundances of bacterial OTUs were merged according to phylum and genus to obtain the top ten phyla and genera in abundance, and the remaining phyla and genera were classified as others. The R package ALDEx2 was used for the significant difference analysis of phylum and genus between groups, and the analysis method was Kruskal–Wallis test. First, we used the aldex.clr function to sample the data based on the Monte Carlo method and obtained the average of the number of Dirichlet instances given by the mc.samples variable. The aldex.kw function was then used for nonparametric ANOVA, with logFC > 1 & *P* < 0.05 as significant enrichment, and logFC < 0.5 & *P* < 0.05 as significant exclusion. The abundance tables classified at the phylum and genus levels were normalized by sample with the R package apply, ggplot2 and ggalluvial were used to draw Sankey diagrams. To ensure aesthetics, the error bars exist only in the lower half of the stacked column, and the phyla and genuses with significant differences (*P* < 0.05) compared to the control group were marked with “*”.

Functional prediction of bacterial communities was performed using FAPROTAX of the Lingbo Microclass Cloud Platform (http://cloud.biomicroclass.com/CloudPlatform/home). Subsequently, multivariate analysis of variance and principal coordinate analysis were performed, and scatter plots were drawn according to the results. The methods were the same as that of adonis analysis, pcoa analysis and mapping based on OTU abundance. Differential analysis and clustering heat map were performed on the functional prediction results of *A. tricolor* and *S. viridis* treated with different treatments respectively, the method was the same as that based on OTU abundance.

## Data Availability

The datasets generated and/or analysed during the current study are available in the BioProject at the National Center for Biotechnology Information (NCBI) repository(https://www.ncbi.nlm.nih.gov/bioproject/?term=PRJNA877400). Project accession number is PRJNA877400.
